# Rac1, A Potential Target for Tumor Therapy

**DOI:** 10.3389/fonc.2021.674426

**Published:** 2021-05-17

**Authors:** Jiaxin Liang, Linda Oyang, Shan Rao, Yaqian Han, Xia Luo, Pin Yi, Jinguan Lin, Longzheng Xia, Jiaqi Hu, Shiming Tan, Lu Tang, Qing Pan, Yanyan Tang, Yujuan Zhou, Qianjin Liao

**Affiliations:** ^1^ Hunan Key Laboratory of Cancer Metabolism, Hunan Cancer Hospital and the Affiliated Cancer Hospital of Xiangya School of Medicine, Central South University, Changsha, China; ^2^ University of South China, Hengyang, China; ^3^ Clinical Research Center for Wound Healing in Hunan Province, Changsha, China

**Keywords:** Rac1, tumorigenesis, metastasis, cancer stemness, therapy resistance

## Abstract

RAS-related C3 botulinum toxin substrate 1 (Rac.1) is one of the important members of Rho GTPases. It is well known that Rac1 is a cytoskeleton regulation protein that regulates cell adhesion, morphology, and movement. Rac1 is highly expressed in different types of tumors, which is related to poor prognosis. Studies have shown that Rac1 not only participates in the tumor cell cycle, apoptosis, proliferation, invasion, migration and angiogenesis, but also participates in the regulation of tumor stem cell, thus promoting the occurrence of tumors. Rac1 also plays a key role in anti-tumor therapy and participates in immune escape mediated by the tumor microenvironment. In addition, the good prospects of Rac1 inhibitors in cancer prevention and treatment are exciting. Therefore, Rac1 is considered as a potential target for the prevention and treatment of cancer. The necessity and importance of Rac1 are obvious, but it still needs further study.

## Background

The classical Rho-GTPase family consists of RhoA (RhoA-RhoC), Rac (Rac1-Rac3 and RhoG), Cdc42 (Cdc42, RhoJ, and RhoQ), and RhoF (RhoD and RhoF) (1). The Rho-GTPase family is involved in a variety of important cellular activities, such as acting skeleton remodeling, cell adhesion, cell movement, vesicle transport, angiogenesis, and cell cycle regulation (2–5). Rho-GTPase family is called “molecular switch” because it can change between the active GTP bound conformation and GDP bound conformation. The activation of the “molecular switch” is controlled by guanine nucleotide exchange factors (GEFs), which stimulates the release of GDP and promote the combination of GTP (6). The inactive state of Rho-GTPase is maintained by the guanine nucleotide dissociation inhibitor (GDIs) and GTPase activating protein (GAPs) (7). With the changes of Rho-GTPase protein level, activity status, and effector protein abundance, the Rho signal becomes abnormal, which may affects the recombination and migration of cells (8). Rac1, Rho, and Cdc42 are the three most important characteristic members of the Rho-GTPase family, and Rac1 has received the most attention (4). Rac1 is widely expressed in tissues, which is considered a regulatory factor related to cell movement and invasion (9). It has been found that Rac1 is highly expressed and over-activated in many cancers. As an intracellular signal transducer, activated Rac1 can control many basic cellular functions, including cytoskeleton dynamics, so as to maintain cell morphology, polarity, adhesion, and migration (10, 11). Unbalanced expression or activation patterns of Rac1 may lead to abnormal cell signal transduction and diseases, such as cancer (4). Drug resistance is the most important reason leading to the poor prognosis of cancer patients. This is due to the formation of a special drug resistance mechanism in tumors, and Rac1 plays an important role in regulating drug resistance in tumor treatment (12). In a word, it is necessary to make a thorough study on Rac1 as an important potential therapeutic target.

## Rac1 Regulates Cell Adhesion, Morphology, and Movement

The Rho-GTPase family (Rho, Rac1 and Cdc42) plays an important role as cytoskeleton regulatory proteins (13), which is best confirmed in fibroblasts and can be observed in many other cell types, such as epithelial cells, endothelial cells, astrocytes and mast cells (14–16). Rac1 regulates cytoskeleton recombination, actin polymerization, and leading-edge extension by promoting actin assembly (17), which is necessary for the formation of lamellar lipid membranes and membrane folding (13). Without Rac1 and Rac2, the ability of osteoclasts to form actin cytoskeleton is insufficient (18).

**Figure 1:**
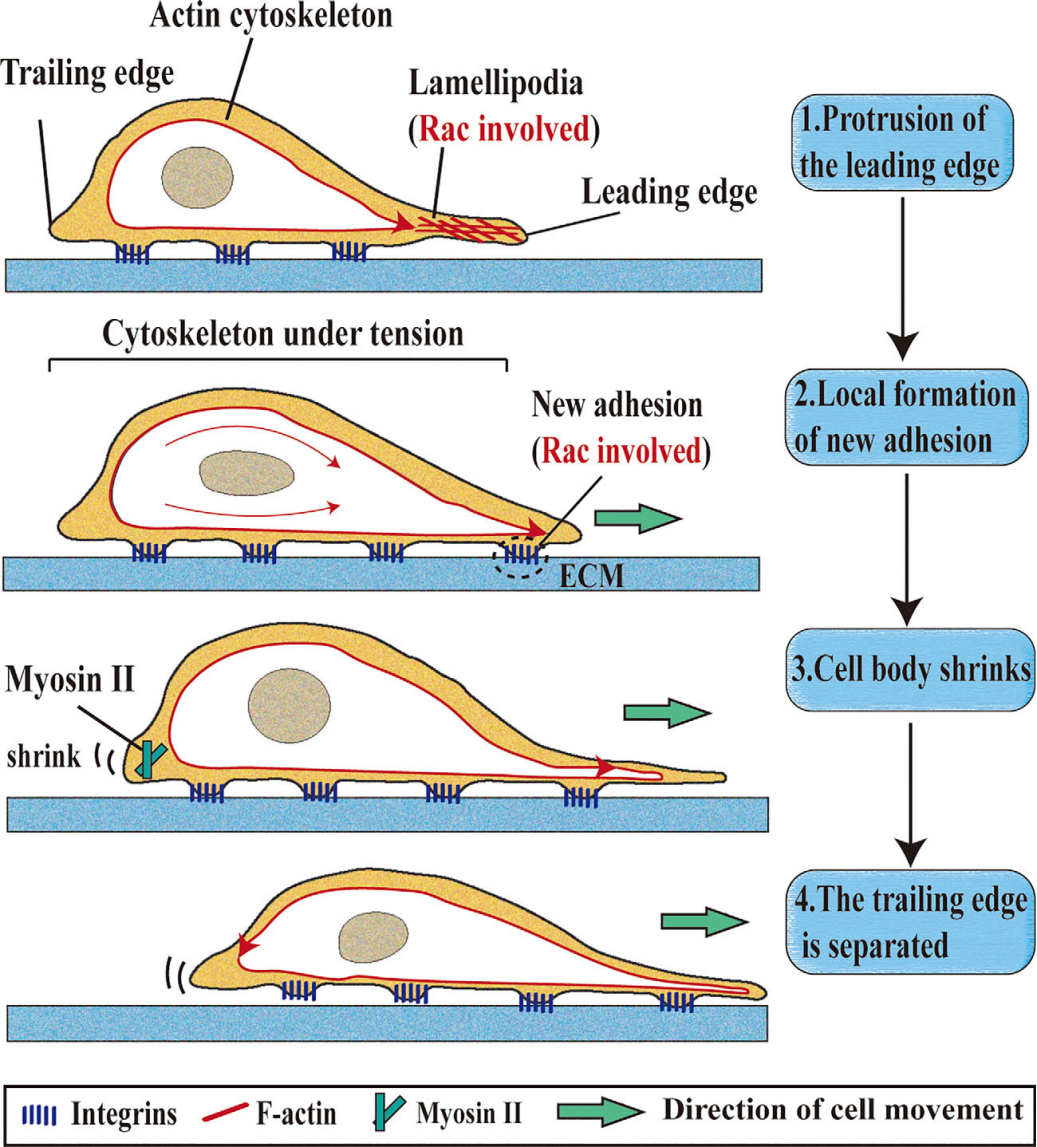
Roles of Rac1 in single-cell migration. Single-cell migration is a multi-step process. 1. Protrusion of the leading edge: Rac1 is located at the leading edge of the cell, remodels the actin cytoskeleton to form lamellipodia, and directs cell migration.; 2. Local formation of new adhesion: integrity contact with extracellular matrix (ECM) ligand and cluster in the cell membrane (Rac1 involved); 3. Cell body shrinks: Myosin II is responsible for shrinking the trailing edge of cells; 4. The trailing edge is separated: The contractile force generated by the actomyosin structure can make the cell movement translation.

Actin polymerization regulated by Rac1 and Cdc42 can promote cell movement, leading to migration and invasion (19). The migration of cancer cells is closely related to the decrease of cell adhesion, rearrangement of cytoskeleton, degradation of the extracellular matrix and the formation of cell surface protrusions, which are the key factor affecting the migration of cancer cells (20). Extension of cytoplasm in the movement direction is the first step of cell movement. In the process of long-distance extension of cells, it is necessary to protrude a wide and flat flaky prosthetic foot on the cell surface, and Rac1 is needed to form this structure (21, 22). The finger-like structures formed in lamellipodium is called filopodia, which is regulated by Cdc42 and participates in cell adhesion (23). Due to different environments, migrating cells present morphological differences (such as expansion, contraction and polarization), which are controlled by the activity levels of cytoskeleton regulatory protein Rho-GTPase (24). CYRI/FAM49B (CYFIP-related Rac1 interacting protein) negatively regulates Rac1-driven cytoskeletal remodeling (25). The dynamic structures of axonal endings in neuroblastoma cell line N1E-115 was activated by Rac1 and Cdc42 (26). The depletion of estrogen receptor α (ERα) affects the biomechanical properties of Breast cancer (BC) cells, which is related to the decrease of cytoskeletal proteins (F-actin, FLNA, and α-tubulin) and cytoskeletal regulatory proteins (Rho, Rac1, and Cdc42) (27). Introducing the mutant Rac1 (P29s) into normal melanocytes can increase membrane folding, and promote proliferation and migration of melanocytes (28). The Rac1 mutant (P29s) melanoma cells can up-regulate the formation of platelet lipoprotein through dendritic actin polymerization (29). When the integrin-associated kinase gene is inactivated in mature melanocytes, motility and dendritic defects occur, which are recovered in the presence of Rac1 (30). Urobilin A (UA), a metabolite of intestinal bacteria, inhibits cell proliferation and migration through destroying the activities of Rac1 and PAK1 (31) (**Figure 1**).

The most mature mechanism of Rac1 mediated cytoskeletal recombination is through PAKs (P21 activated kinase) (32). Both cell proliferation and cell movement require actin recombination, which is controlled by Rac1 and PAK1 (31). PAKs can be divided into two groups: one is PAK 1-3 and the other group is PAK 4-6 (33). The C-terminal kinase domain of PAK subgroup I is a highly conserved sequence, which can exert its biological activity by binding Cdc42 or Rac1 (34). PAK1 is an important downstream effector of Rac1 and Cdc42 (35). Rac1 and Cdc42 can activate LIM kinase1 (LIMK1) through PAK, which leads to the decrease of cofilin activity and enhance mobility through phosphorylation (36). The effect of PAK on cell mechanics depends on Rac1, and the formation of the Rac1-PAK pathway plays an important role in cytoskeleton reorganization during cell migration (19). Abnormal high expression of non-receptor tyrosine kinase FER is the key to metastasis of ovarian tumor cell *in vitro* and *in vivo* (37). When FER is knocked out, the Rac1-PAK1 signaling pathway is inactivated and the migration ability of ovarian cancer cell CAOV4 decreased (38). Knocking down PKC-can lead to a decrease in the proliferation and metastasis of colorectal cancer (CRC) cells because PKC-ζ reduces the nuclear translocation of β-Catenin and affects the Rac1-PAK1-β-catenin signaling cascade (39).

## The Expression and Clinical Significance of Rac1 in Tumors

**Figure 2:**
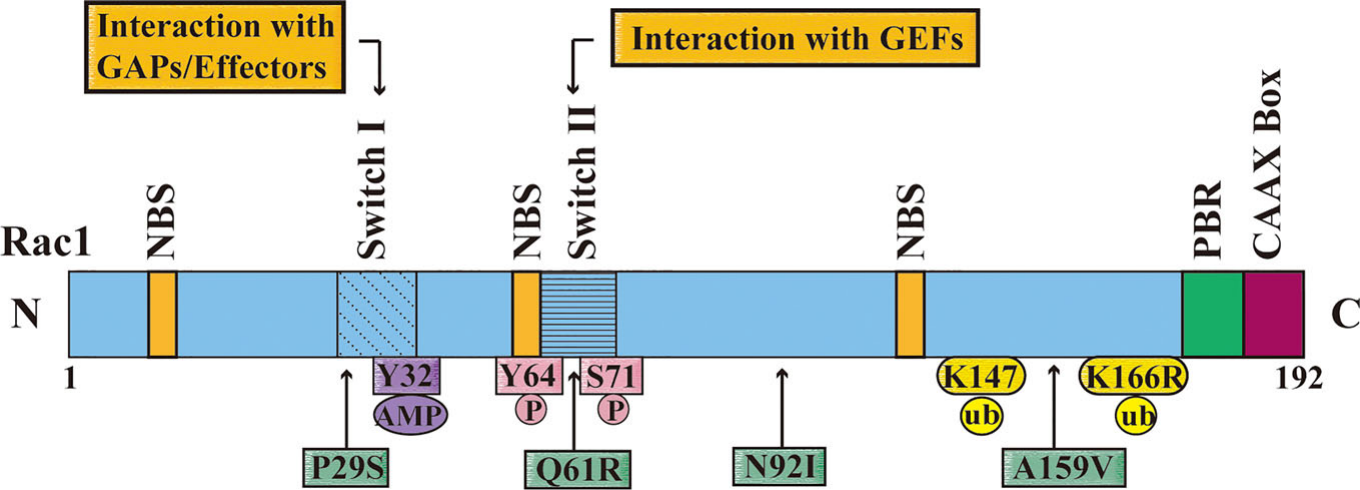
The domain, post-translational modifications and mutation sites of Rac1. The figure shows that the different domains of Rac1 including nucleotide-binding site (NBS), switch I, switch II, multi-base region (PBR), and CAAX box. Switch I mainly interacts with the downstream effectors of RAC1, and Switch II interacts with the RAC1 activation protein guanine nucleotide exchange factor (GEF). The figure also shows the Rac1 adenylation site (Y32), phosphorylation sites (Y64 and S71), and ubiquitination sites (K147 and K166); and important missense mutations of Rac1 (P29S, Q61R, N92I and A159V) are displayed with a green frame, and the position is pointed out with an arrow.

The malignant transformation of tumor is mainly related to over-activation or over-expression of Rac1. Up to now, the increase of Rac1 expression has been detected in different types of cancers, such as BC, lung cancer, colorectal cancer, gastric cancer, prostate cancer, hepatocellular carcinoma and ovarian cancer (40–46). The activity of Rac1 is also related to many post-translational modifications, such as phosphorylation (Tyr64, Ser71), ubiquitination (Lys147, K166R), lipidation, and adenylation (Y32) (47–53). Rac1 usually does not mutate, except in certain cancers, such as melanoma. Rac1 (P29S) is the third most common mutation codon in human skin melanoma, affecting 4-7% of patients (28) (**Figure 2**).

The mutant form of Rac1 (N92I) found in human sarcoma cell line HT1080 not only makes Rac1 highly carcinogenic, but also resists endoplasmic reticulum stress (54, 55). The mutants of Rac1 (P29S) or (N92I) can improve the level of the active binding status (Rac1-GTP) by promoting the decomposition of the intrinsic inactive binding status (Rac1-GDP) of Rac1, thus forming a “spontaneous activation” state, which strongly promotes the occurrence of tumors (54, 56). Other forms of Rac1 mutants are also found in other tumors, such as the Rac1 (A159V) mutation common in Head and Neck Neoplasms and the Rac1 (Q61R) mutation in primary prostate cancer (57, 58). The expression of Rac1 protein in different tumor tissues was detected by immunohistochemistry. It was found that the high expression of Rac1 protein was closely related to the differentiation, staging and lymph node metastasis of tumor (59, 60). The analysis and detection of renal cell carcinoma(RCC) samples showed that Rac1 protein was highly expressed, which was positively correlated with the poor clinical prognosis of RCC (61). The expression of Rac1 in gastric cancer tissues was detected, and it was found that the expression of Rac1 in TNM III and IV stages was higher than that in I and II stages and was associated with tumor lymph node metastasis (62). When epithelial ovarian cancer, primary gallbladder cancer (PGC) and hepatocellular carcinoma are analyzed, the same conclusion is reached (40, 63, 64). According to bioinformatics analysis, it was found that the overexpression of Rac1 and RRM2 was closely related to the poor prognosis of HER-2 positive BC patients (41). In the analysis of miRNA related to gastric cancer metastasis, it was found that the high level of miR-345 was positively correlated with the good prognosis of patients, because miR-345 down-regulated the transmission of epidermal growth factor receptor pathway substrate 8 (EPS8) and Rac1 signal (65). HACE1 is a ubiquitin ligase of E3. In lung cancer and prostate cancer, the low expression of HACE1 can make Rac1 overactive, which is related to the shortened survival time of tumor (66, 67). A meta-analysis shows that high expression of Rac1 could predict the poor prognosis of cancer patients (68). Therefore, abnormal expression of Rac1 can be used as a monitoring index for the progression and poor prognosis of different types of cancers.

## Regulation of Rac1 in Tumor Progression

The inactive state of Rac1 can combine with various effector proteins and regulate cell events, such as cell cycle, cell proliferation, apoptosis, stem cell characteristics of cancer cells, and neovascularization, thus participating in the occurrence and development of cancer (3, 56, 69, 70).

### Rac1 Participates in the Cell Cycle, Apoptosis, and Proliferation

Rac1 shuttles between cytoplasm and nucleus during the whole cell cycle, and accumulates in nucleus in the late G2 stage (71). And the induction of the G1 cell cycle by Rac1 is independent of the JNK/SAPK MAP kinase cascade (72). Rac1 overexpression activates the 70kDa ribosomal S6 kinase (pp70S6k), which plays an important role in the G1 phase of the cell cycle (73). Overexpression of ARHGAP24 significantly inhibits the activities of RhoA and Rac1 and induced apoptosis of lung cancer cells *via* STAT6-WWP2-p27 axis (74). Rac1 participates in the formation and elimination of apoptotic cells and coordinates receptor signals related to apoptosis and proliferation (75, 76). In B-cell lymphoma, Rac1 was found to be a new binding partner of Bcl-2, which can stabilize its anti-apoptosis activity (77). It has been confirmed that the regulation of chromosome condensation 2 (RCC2) on apoptosis is mediated by inhibiting Rac1 signal transduction (78). Rac1 can also form miR-506-rock2-rac1 signal axis with mir-506 and ROCK2 (Rho protein kinase 2), and participate in the proliferation and apoptosis of hepatocellular carcinoma (HCC) cells (79). The apoptosis of glioma cells induced by Rac1 inhibition can be partly saved by mitogen-activated protein kinase 1, which is the activator of JNK (80). Lionarons et al. further revealed that Rac1 (p29s) can activate the gene expression program initiated by the PAK, AKT and SRF/MRTF transcription pathways. It can induce melanocytes to transform into mesenchymal-like cells, inhibit apoptosis, and enhance tumorigenesis (81). OPA interacting protein-5 (OIP5) regulates proliferation, apoptosis and cell cycle of HCC cells by influencing BMPR2-JUN-CHEK1-Rac1 signal axis (82).

### Rac1 Promotes Tumor Angiogenesis

Angiogenesis is one of the hallmarks of malignant tumors. Overexpression of Rac1 is related to the high levels of vascular endothelial growth factor (VEGF) and vascular endothelial growth factor receptor (VEGFR) can form a VEGF-VEGFR signaling pathway, which participates in the regulation of angiogenesis (83, 84). The role of Rac1 in retinal angiogenesis has been well documented (85, 86). IQ- guanosine triphosphatase activating protein 1 (IQGAP1) is a scaffold protein with Rac1 binding domain, and its knock-out can significantly inhibit choroidal neovascularization induced by the VEGFR2-Rac1 signal axis (87). Sphingosine-1 phosphate receptor1 (S1PR1) can amplify the angiogenic signal of VEGF-VEGFR2, thus maintaining the activity of Rac1 and promoting the growth of tumor (88). Sevoflurane, a volatile anesthetic agent, exerts an anti-angiogenic effect by inhibiting the signal transduction of Rac1-paxillin-FAK and Ras-Akt-mTOR (89). M1 macrophage-derived exosomes (M1-Exos) inhibit the Rac1-PAK2 signaling pathway and decrease the angiogenesis ability of endothelial cells (ECs) (90). In mouse models, it has been found that the activation of PAK1 by endothelial Rac1 is helpful for post-stroke recovery and angiogenesis (91). NCK1, an adaptor with Src homologous domain, can promote the angiogenesis of cervical squamous cell carcinoma (CSCC) through the Rac1-PAK1-MMP2 signaling pathway (92). In the study of aristolochic acid-induced nephropathy, it was found that over-expression of NCK1 can not only restore the decrease of Rac1 activation, but also save the damaged angiogenesis (93). Tomm7 (translocase of outer mitochondrial membrane 7) gene is selectively inactivated, which induces an increase in the entry of Rac1 into mitochondria, and promotes the redox signal transduction coupled with mitochondria Rac1, which leads to cerebrovascular disorders and affects the homeostasis of the cerebrovascular network (94).

### Rac1 Participates in Tumor Migration and Invasion

Overexpression of Rac1 enhances cell proliferation and migration, and plays an important role in the invasion and migration of many tumor cells (65, 95–99). Atypical protein kinase C-ζ (PKC-ζ) mediates BC cell invasion through Rac1 and RhoA pathways (100). A high concentration of stromal cell-derived factor 1-α (SDF-1α) promotes the expression of Rac1 and mediates the migration and adhesion of BC cells (101). The ability of the oral contraceptive centchroman (CC) to inhibit migration and invasion of BC cells is achieved by inhibiting the Rac1-PAK1-β-catenin signal axis (102). The stimulation of NF-κB by Rac1 partly regulates the proliferation and invasion of the melanoma cell line FEMX (103). Our research team found that Rac1 was significantly up-regulated in metastatic colorectal cancer tissues, and the overexpression of Rac1 can significantly promote the migration and invasion of colorectal cancer cells (43). We have further found that diallyl disulfide (DADS) inhibits the migration and invasion of colorectal cancer cell line SW480 by regulating the Rac1-ROCK1/PAK1-LIMK1-ADF/cofilin signaling pathway (104, 105). Zhang, et al. found that Plastin1 (PLS1), which is related to the microvilli structure of the intestinal epithelium, drives the metastasis of colorectal cancer through the IQGAP1-Rac1-ERK pathway (106). Overexpression of phospholipid phosphatase-related protein 1 (PLPPR1) in the mouse neuroblastoma cell line (Neuro2a) reduces the level of active Rac1. Therefore, it can lead to increased cell adhesion and decreased cell migration (107). miR-142-3p inhibits the migration of bladder cancer cells through Rac1 (97). The long non-coding RNA LCAT1 is a competitive endogenous RNA of miR-4714-5p, which leads to the up-regulation of the activity of its endogenous target Rac1 and promotes the growth and invasion of lung cancer cells (96).

Epithelial-mesenchymal transition (EMT) is another important characteristics in the process of tumor cell metastasis. Cells undergoing EMT will lose their cell polarity and adhesion between cells, which is related to the reorganization of the cytoskeleton structure (108). Rab23 is a member of the Ras-related small GTPase family, which can activate Rac1-TGFβ signal transduction and promotes EMT in HCC cells (109). POTEE, a member of the POTE anchor protein family E, promotes invasion and migration of colorectal cancer and EMT by promoting the activation of Rac1 and Cdc42 (110). miR-331-3p targets ErbB2 and Vav2 through the Rac1-PAK1-β-catenin axis to inhibit EMT, migration and metastasis of non-small cell lung cancer (NSCLC) cells (111). Tualang honey in the jungle of Malaysia maintains the epithelial polarity of cells by overexpressing β-catenin and E-cadherin, and inhibits the invasiveness of oral squamous cell carcinoma (OSCC) by downregulating TWIST1 and Rac1 (112). Our previous experiments confirmed that Rac1 can influence the expression of EMT-related molecules, and participate in the invasion and metastasis of CRC (43). Guanine nucleotide exchange factor T (GEFT) affects the occurrence of EMT and interstitial transformation (MET) in rhabdomyosarcoma (RMS) cells through the Rac1-Cdc42-PAK1 (113). miR-142-3p affects the expression of Rac1 at the protein level, thus inhibiting the phosphorylation of PAK1 and EMT in BC cells (114).

### Rac1 Functions in Cancer Stem Cells

The stemness of tumor cells is considered to be a key factor in tumor initiation, progression, and recurrence. Rac1 is involved in the regulation of stem cell characteristics of various tumor cells. Rao, et al. found that Rac1 participates in semaphorin-3F (Sema3F) -mediated CRC cell stemness regulation by targeting the classical Wnt-β-catenin pathway (115). Rac1 can participate in the regulation of intestinal stem cell proliferation and the occurrence of colorectal cancer through the activation of Wnt pathway by NF-κB or in a ROS-dependent manner (116, 117). Integrin can activate the Rac1 signaling pathway in stem cells, and thus stimulate Wnt pathway. Integrin-β1/Rac1 signal plays an important role in the maintenance and self-renewal of mammary epithelial stem cells (118). The expression of integrin-α5 (ITGA5) is down-regulated by miR-205, which can inhibit the stem cell characteristics of triple negative breast cancer (TNBC) through the Src-Vav2-Rac1 pathway (119). However, Carmon, et al. found that LGR5 (containing the leucine-rich repetitive sequence of G protein-coupled receptor 5) mainly activates the IQGAP1-Rac1 pathway, but not Want signaling pathway, so as to promote cell adhesion between stem cells and colon cancer cells (120). LncRNA NR2F2-AS1 mediated the up-regulation of Rac1 expression can increase the cancer stemness of clear cell renal cell carcinoma (ccRCC) cells (121). Inhibition of β2-chimaerin protein mediated by hippocampus effector TAZ leads to the persistence of Rac1 activity in cancer stem cells (CSCs) (122). Semaphorin-3C (Sema 3C) is involved in promoting the survival and tumorigenicity of glioma stem cells by activating Rac1, which is related to activating Rac1-NF-κB signal (123, 124). Inhibition of Rac1 can block the proliferation and metastasis of NSCLC tumor stem cells (125). Both miR-365 and miR-194 can inhibit the dedifferentiation of HCC cells and the proliferation of HCC stem cells by targeting Rac1 signals (126, 127). In addition to endowing tumor cells with the ability of migration and invasion, EMT can also make highly invasive tumor cells acquire stem cell-like characteristics and promote the production of CSCs (128). In NSCLC, inhibiting Rac1 activated during EMT can inhibit the dynamic transformation between cancer stem/progenitor cells (CS/PC) and non-CS/PC (129). DJ001 is a receptor-type protein tyrosine phosphatase-sigma (PTPσ) inhibitor, which can inhibit radiation-induced apoptosis of hematopoietic stem cells (HSCs), and promote the regeneration of HSC by activating Rac1 and inducing the expression of Bcl-xl (130).

**Figure 3:**
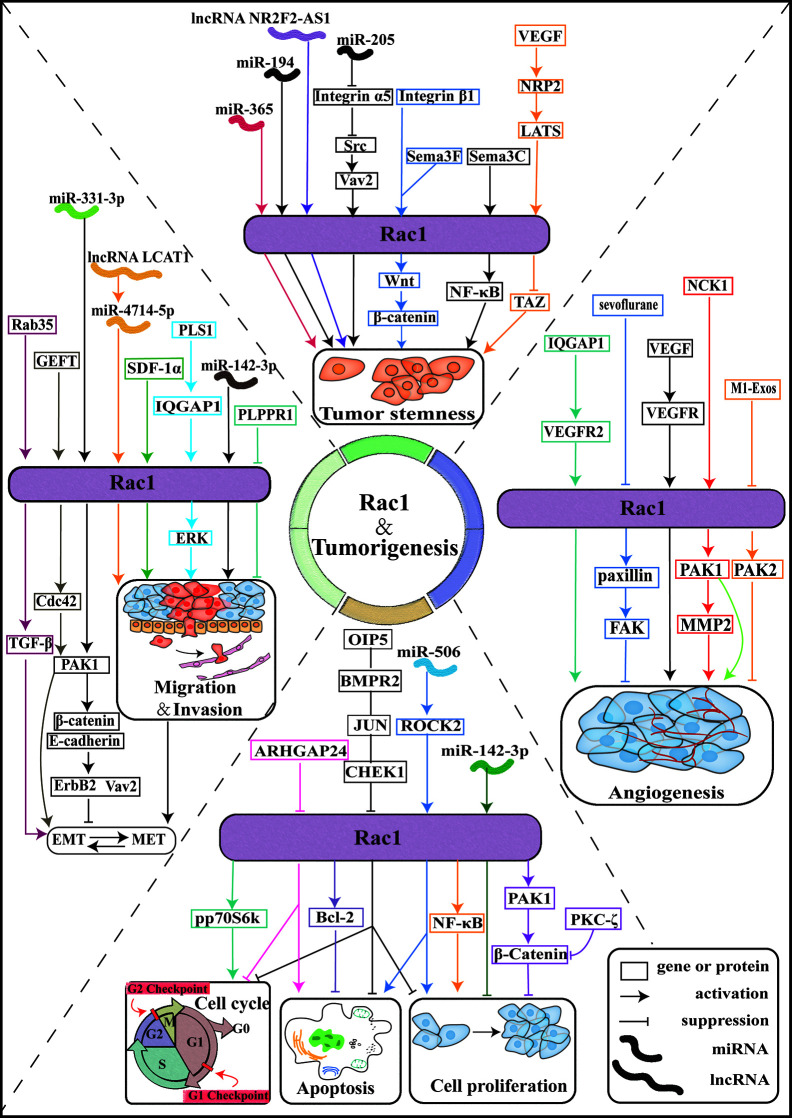
Rac1 is involved in tumorigenesis. Schematic diagram of the Rac1 signaling pathway and effectors. The Rac1 signaling pathway plays an important role in the pathobiology of various tumor progression processes, including tumor cell proliferation, cell cycle, apoptosis, tumor cell invasion, and migration, tumor angiogenesis, and tumor cell stemness. ARHGAP24: Rho GTPase activating protein 24; PKC-ζ: protein kinase C-ζ; PAKs: p21 activated kinase; M1-Exos: M1-type macrophage-derived exosomes; IQGAP1:IQ-guanosine triphosphatease-activating protein 1; SDF-1α: stromal cell-derived factor 1-α; Sema3F: semaphorin-3F; Sema3C: Semaphorin-3C.

In recent years, the mechanism of Rac1 involved in the regulation of different tumorigenic phenotypes has been elucidated. As an important goal of cancer prevention and treatment, the relationship between Rac1 and tumorigenesis, proliferation, metastasis, and development of drug resistance has gradually become clear. Although we already know that Rac1 plays an important role in cancer, there are few clinical studies related to Rac1, and the detailed mechanism of Rac1 participating in cancer has not yet been clarified. The synergistic effect of Rac1 and other carcinogens is particularly reflected in the intersection of signal pathways, which needs further exploration (**Figure 3**).

### Rac1 Is Involved in the Regulation of Resistance to Tumor Therapy

The application of molecularly targeted drugs has greatly improved the clinical efficacy of cancer treatment. However, with the progress of treatment, acquired drug resistance appears, which greatly reduces the treatment effect and even leads to failure. The development of molecular targeted drug resistance is closely related to cancer as a dynamic and highly heterogeneous disease. This is because the heterogeneity of cancer not only drives the development of cancer, but also affects drug resistance, which provides a driving force for the drug resistance of cancer treatment (131, 132). In addition, mutations of the drug target gene and the enhancement of DNA damage repair ability are also the important mechanisms for drug resistance (133, 134). Therefore, the analysis of the mechanism of drug resistance is helpful to find effective targets to overcome drug resistance, to better select effective treatment methods for patients and to improve prognosis.

The increase of Rac1 expression is one of the characteristics of drug-resistant cells (135). The instability of the genome leads to an increase in mutation rate, and then promotes the development of cancer, which affects drug resistance through various mechanisms. Taking melanoma as an example, Rac1 is an important somatic-driven mutation gene in melanoma (136). Melanoma is mostly treated with inhibitors of the MAPK signaling pathway that targets BRAF or MEK kinase (137). However, the treatment response rate in the middle and late stages of treatment is not high, which may be due to the reactivation of the MAPK signaling pathway and/or the activation of the PI3K-AKT pathway caused by selected genetic changes before or during treatment, which caused the primary and acquired resistance (138). BRAF600E and Rac1P29S are hot spot mutations in melanoma. MAP kinase pathway inhibitor (MAPKi) is effective for melanoma patients with BRAFV600E mutation. According to the understanding of MAPKi resistance mechanism, it was found that CUL3, the key protein in the E3 ubiquitin ligase complex, participated in vemurafenib resistance mechanism by enhancing Rac1 activity and MEKS 298 phosphorylation. Furthermore, it was found that the Src family inhibitor Saracatinib can inactivate Rac1, thereby inhibiting MAPKi resistance phenotype (139). Rac1P29s was found to confer resistance to BRAF/MEK inhibitors (BRAFi/MEKi) to melanoma cells (140), which can be reversed by SRF/MRTF inhibitor (81). When drug resistance occurs in tumors, the endogenous metabolic profile changes significantly, and Rac1 is involved in it. Li, *et al.* showed that targeting Rac1 can effectively reduce the multidrug resistance of BC cells to neoadjuvant chemotherapy (NAC). This is because Rac1 activates aldolase A (ALDOA) ​​and ERK signaling, thereby up-regulating glycolysis, especially the non-oxidized pentose phosphate pathway (PPP), which leads to the enhancement of nucleotide metabolism (141). Rac1 silencing can also inhibit AKT-FOXO3a signal and cell glycolysis enzymes, so as to overcome cisplatin resistance in esophageal squamous cell carcinoma (ESC) (142).

The expression of Rac1 regulates the sensitivity of cancer to chemotherapy (143). Targeting the Rac1 pathway can overcome the resistance of NSCLC patients to EGFR-TKI, and it works independently of the MEK or PI3K mechanism (144). Silencing of cadherin 2 (DSG2) can inhibit the EGFR-Src-Rac1-PAK1 signaling pathway and increase resistance to osimertinib (145). In multidrug-resistant lymphoma cell lines expressing a higher levels of Tiam1, the researchers found that dual inhibition of Tiam1-Rac1 and Notch pathways would be an important treatment for overcoming the resistance of lymphoma cells to adriamycin (146). Hofbauer, et al. found that inhibition of Tiam1-Rac1 signaling can antagonize the chemical resistance of chronic lymphocytic leukemia (CLL) cells to fludarabine (147). YAP, a key effector of the Hippo pathway, confers multidrug resistance to HCC cells by up-regulating the Rac1-ROS-mTOR pathway, which leads to the inhibition of autophagy-related cell death (148). Similarly, Rac1 also affects the sensitivity of cancer to radiotherapy. RP-4, a new type of radiosensitizer derived from rhein, activates the sensitivity of nasopharyngeal carcinoma (NPC) cells to radiotherapy by targeting the Rac1-NADPH pathway (149). Our research team has confirmed that Rac1 can target the PAK1-LIMK1-Cofilins signaling pathway to cause radiotherapy resistance in lung cancer (150). In the treatment of head and neck squamous cell carcinoma (HNSCC), it was found that the combination of Rac1 inhibitors based on radiotherapy can improve the therapeutic effect (151). Inhibition of Rac1 with specific inhibitors of Rac1 not only eliminates the activation of G2 checkpoints induced by radiotherapy (IR) but also improves the sensitivity of pancreatic cancer cells to radiotherapy by inducing apoptosis (152). It is evidenced that inhibition of Rac1 activity can be used to overcome treatment resistance, which has also been confirmed in cisplatin-resistant gastric adenocarcinoma cells (153) and trastuzumab-resistant BC cells (154).

**Figure 4:**
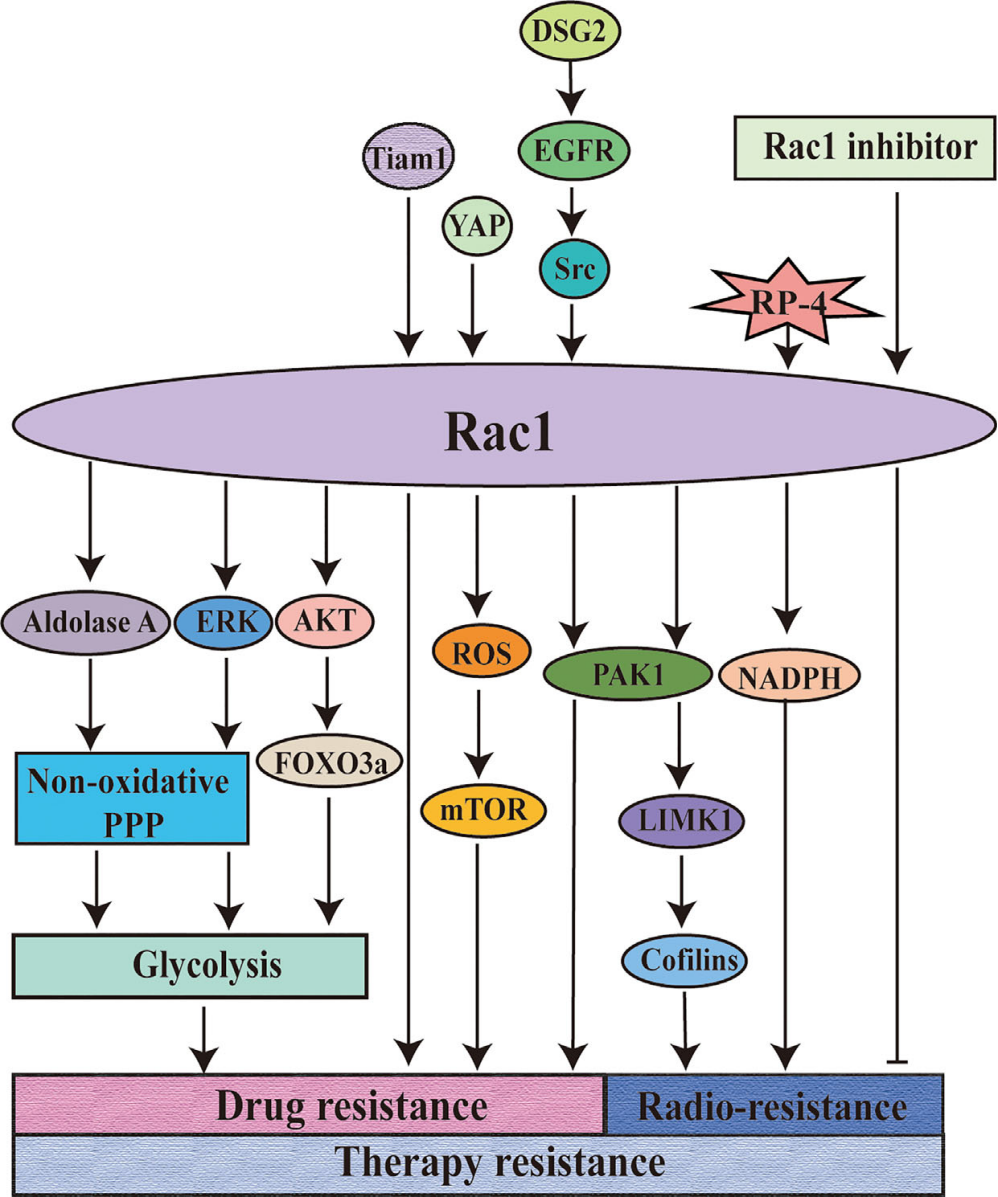
Rac1 is involved in the regulation of resistance to tumor therapy. Rac1 is involved in the regulation of tumor treatment resistance. Targeting Rac1 can improve the sensitivity of the tumors to chemotherapy and overcome the drug resistance of the tumors to molecularly targeted drugs. The combined application of Rac1 inhibitor on the basis of radiotherapy can significantly improve the therapeutic effect. DSG2: cadherin 2.

As shown before, Rac1, as an important “commander” of drug resistance in tumor therapy, regulates the drug resistance of tumor cells to targeted drugs by participating in various mechanisms and thus affects the sensitivity of tumor cells to radiotherapy and chemotherapy. However, although Rac1 may be a useful target for overcoming drug resistance, the mechanism of each has not been clearly explained (136, 155), and it is necessary to explore how Rac1 participates in the resistant mechanism. It is worth mentioning that the regulatory mechanism of Rac1 on the endogenous metabolic profile in the development of drug resistance can be further explored. Using evolutionary thinking to deal with drug resistance is an important way to deal with heterogeneity and evolutionary drug resistance of tumors. It is also worth noting that the combined treatment of targeted Rac1, radiotherapy, and chemotherapy combined with Rac1 inhibitor may be a reasonable and reliable solution to improve the sensitivity of tumor cells to radiotherapy and chemotherapy and overcome the drug resistance of tumor cells to targeted drugs (**Figure 4**).

### Rac1 Participates in Tumor Microenvironment-Mediated Immune Escape

Tracing back to the “seed and soil” hypothesis published by Paget in the 19th century, we know that the occurrence and development of tumors depend not only on the tumor cells themselves but also on the environment in which tumors depend, that is, the tumor microenvironment. The tumor microenvironment is mainly composed of various infiltrating immune cells and other interstitial cells, as well as a variety of secretory factors. With the development of the tumor, the tumor microenvironment changes the immune microenvironment by recruiting or amplifying a variety of immune heterogeneous cells, blocking the effective immune monitoring ability of hosts and mediating immune escape.

There is some correlation between Rac1 and immunity (156, 157). Rac1 regulates the immune homeostasis of the liver, which may exist as an immune checkpoint (158). The activation of Rac1 can drive the pathogenic interaction between epithelial cells and the immune system, which is also the pathological basis of psoriasis (159). Rac1 and Rac2 play a role in the regulation of B cell humoral immune response and *in vitro* Ig class switching (76). Rac1 forms a complex with Tiam1 and regulates the transcription of interleukin 17A (IL17A) and autoimmunity (160). TNFAIP8L2/TIPE2 (tumor necrosis factor, alpha-induced protein 8-like 2) can directly bind to and block Rac1 GTPase activity, thereby regulating innate immunity (161). PI3K activates immunity by up-regulating IL-10 and inhibiting pro-inflammatory cytokines, possibly through the regulation of Rac1 protein (162). Withanolides, as immunopotentiators and antiviral agents against COVID -19, may play a role by targeting the Rac1 protein (163). Compared with Rac1 WT, melanoma cells with Rac1(P29S) mutations express high levels of PD-L1 and therefore have the ability to evade immune surveillance (164). The use of lenalidomide, an immunomodulatory drug, can restore the normal activity levels of Rac1, RhoA, and Cdc42 in T cells of patients with chronic lymphocytic leukemia (CLL), and preserve the adhesion and movement ability of T cells and the function of integrin lymphocyte activation-related antigen 1 (LFA-1) (165). Therefore, targeted inhibition of Rac1 in tumor cells may be a way to prevent immune escape, and may also be used as immune checkpoint inhibitors (ICIs).

## General Rac1 Inhibitor

Guanine nucleotide exchange factors (GEFs) are responsible for the activation of Rac1. Up to date, more than 70 GEFs related to Rac1 activation have been confirmed. GEFs are related to tumorigenesis, but the specific regulatory mechanism is still unclear, and different GEFs have different abilities to activate Rho family GTPases. The Rac1 inhibitor developed for the interaction between GEF and Rac1 is an important strategy for finding clinical drug candidates. As an inhibitor of Rac1, its most essential role is to inhibit the tumorigenicity mediated by Rac1 and the malignant biological behaviors of different tumor cells, such as cell migration, cell invasion, and rearrangement of actin skeleton (166, 167).

As the first specific Rac1 inhibitor, NSC23766 targets Rac1 activation through GEFs (Trio or Tiam1) but does not interfere with the binding or activation of the closely related targets Cdc42 or RhoA (168, 169). The seven residues on Rac1 are very important for the interaction of NSC23766 and Tiam1 (170). It has been found that NSC 23766 can inhibit the invasion and migration of human HCC by inhibiting the CAMSAP2-dependent Rac1/JNK pathway, or the cysteine-rich domains-1(LMCD1)-Rac1 pathway (171, 172). The inhibition of Rac1 by NSC23766 can regulate NF-κB activity, cell proliferation, and cell migration in NSCLC cells (166). EHop-016 is a derivative of NSC23766. Compared with the parental compound, EHop-016 has a lower IC_50_. EHop-016 inhibits the binding of active Vav2 to the nucleotide-free Rac1 (G15A) mutant fusion protein and inhibits the activity of the downstream effector p21 activated kinase (PAK), valve foot extension, and cell migration in metastatic cancer cells (173). EHop-016 can also reduce the activity of Akt and Jun kinase (JNK) and the expression of c-Myc and Cyclin D, and increase the activity of caspase 3/7 in metastatic cancer cells, thereby affecting cell survival (174, 175). EHop-016 can eliminate the growth, metastasis, and self-renewal ability of gallbladder cancer cells caused by overexpression of miR-365 (176). Unlike NSC23766, EHT1864 was initially described as a small molecule, which interfered with the binding of nucleotides to Rac1 and prevented the binding of GTPase to downstream effectors (177, 178). The growth inhibition of BC cells induced by EHT1864 is related to the dual inhibition of PI3K-AKT-mTORC1 and MEK-ERK pathways (179). Studies have shown that Rac1 plays an important role in the contraction of various smooth muscles, such as bronchial smooth muscle, bladder smooth muscle, and prostate smooth muscle (180–183). Although both EHT1864 and NSC23766 are Rac1 inhibitors, they have different pharmacological mechanisms in the inhibition of different smooth muscles (182, 184, 185), and EHT1864 tends to perform better (186, 187). In recent years, new Rac1 inhibitors have been continuously developed. ZINC69391 is a specific Rac1 inhibitor, which interferes with Rac1-GEF interaction by masking Trp56 residue on Rac1 surface. The 1A-116 analog is a Rac1 inhibitor designed and developed based on ZINC69391. It plays a role by inhibiting the interaction of Rac1 with the Vav family (Vav1-3), Tiam1, and Dbl (188). Trp56 is necessary for 1A-116 to play an inhibitory role (189). ZINC69391 and 1A-116 can inhibit the interaction of Rac1 and GEF (Tiam1, Dock180) (188, 190, 191). It has been found that 1A-116 and its parental compound ZINC69391 can inhibit the proliferation, invasion, migration, and cell cycle of BC cells, glioma cells, and leukemia cells, and 1A-116 shows higher specificity and intensity *in vivo* and *in vitro* (190, 191). 1D-142 is a newly discovered new guanidine inhibitor, which can inhibit the activation of Rac1 by interfering with the interaction of Rac1-Tiam1, and its effectiveness *in vivo* and *in vitro* is much higher than that of the reported derivative 1A-116 (192). In HCC mouse model, 1D-142 was found to significantly reduce tumor growth and intrahepatic metastasis (193). Similarly, in mouse models of NSCLC, 1D-142 was found to inhibit NSCLC cell proliferation and migration by reducing Rac1-mediated TNFα-induced NF-κB nuclear translocation (192).

In the process of exploring Rac1 inhibitors, there are many gratifying discoveries: for example, the inhibitory effect of NSC23766 on Rac1 can antagonize the drug resistance of tumor cells to targeted drugs, such as fludarabine resistance of chronic lymphocytic leukemia (CLL) cells (147), fluorouracil and cisplatin resistance of gastric adenocarcinoma cells (153), and trastuzumab resistance of BC cells (154). NSC23766 increases the anti-proliferative effect of erlotinib on glioblastoma cells in a synergistic manner (167). EHop-016 was found to be an effective inhibitor of human and mouse leukemia cells, but NSC23766 does not have the ability, which may be related to the specific targeting of EHop-016 to Vav1 (194). ZINC69391 and 1A-116 can selectively induce apoptosis of leukemia cells from patients, which may be related to significant activation of caspase-3 and loss of mitochondrial membrane potential (191). This suggests that the combination of drugs targeting Rac1 and other therapeutic drugs may provide a new direction for the treatment of leukemia. EHT1864 can inhibit the proliferation of BC cells induced by increased transcription activity of estrogen receptor-α (Erα) (195). The pharmacological inhibitory effect of 1A-116 on the Rac1-PAK1 axis, inhibition of PAK1 activity and reduction of the level of estrogen receptor phosphorylation at Ser305, is beneficial to the recovery of drug resistance mechanisms in endocrine therapy (196). Gonzalez, et al. confirmed that 1A-116 can also inhibit the Rac1 (P29S) mutation of melanoma (189). EHT1864 can block the Rac1-dependent processing of amyloid precursor protein, and amyloid precursor protein forms senile plaque of Alzheimer’s disease (AD), which provides a prototype for developing new drugs suitable for AD therapy (197). EHT1864 can reduce kidney injury caused by salt and aldosterone by abnormal activation of the mineralocorticoid receptor (MR) mediated by Rac1 (198). Ziegler, et al. also confirmed that the use of EHT1864 can promote DNA repair and reduce DNA damage induced by radiotherapy (IR) (199). Rac1 inhibitors are considered to be beneficial in the treatment of many diseases, but at the same time, some scholars have proposed that the widespread use of Rac1 inhibitors (such as NSC23766 and EHT1864) may cause serious off-target effects in mouse platelets (200). It is interesting to note that Xie, et al. found that deacetylepoxydiene (DA-MED) acts as a Rac1 agonist in human NSCLC H1299 cells with p53 gene deletion and also activates the massive production of ROS (201).

**Figure 5:**
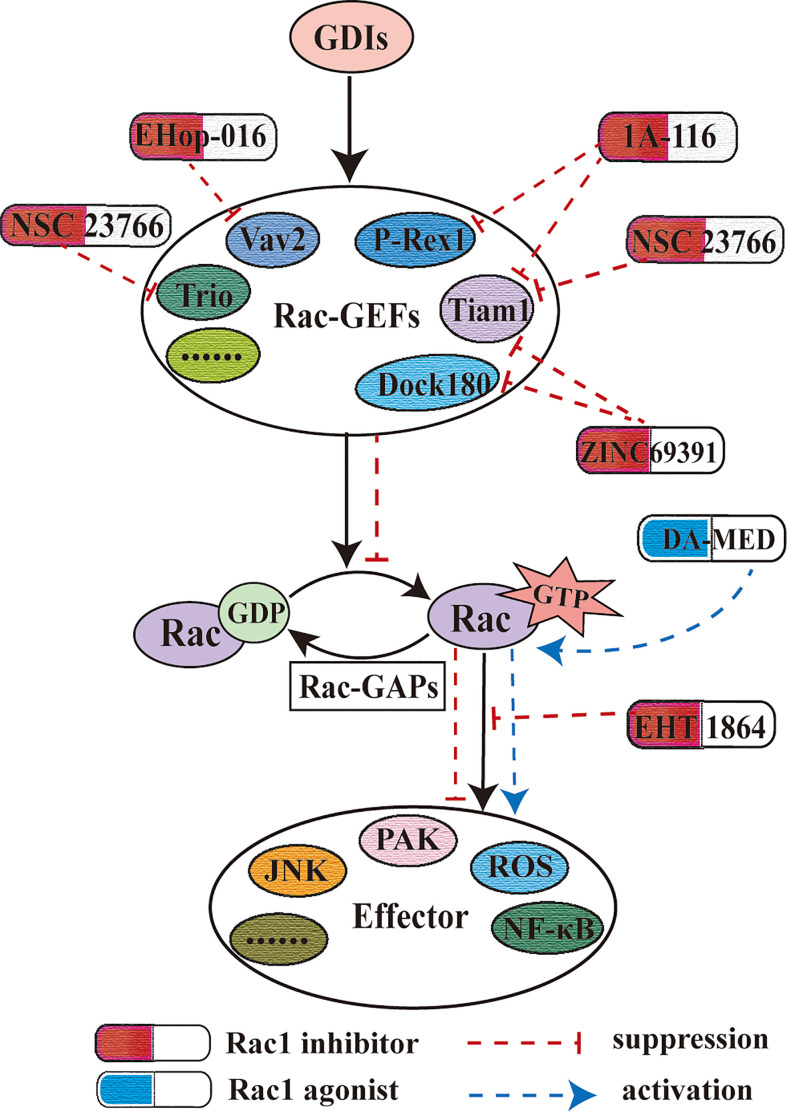
Rac1 inhibitor and Rac1 agonist. Rac1 inhibitors play a role by blocking the activation of Rac1 by guanine nucleotide factor, the upstream effector of Rac1. This figure also shows the effect of the different Rac1 inhibitors mentioned herein on Rac1-GEF.

It is worth noting that Rac1 inhibitors have made some breakthroughs in the clinical applications. R- ketorolac, as a component of a racemic drug approved by the FDA to relieve pain, is a dual Rac1/Cdc42 inhibitor, which can reduce the invasive biological behavior of ovarian cancer and glioblastoma (202–204). A novel anthraquinone−quinazoline hybrid 7B blocks TNBC cell migration, invasion and EMT *via* targeting EGFR and Rac1 (205). However, the limitations of the clinical application of Rac1 inhibitors cannot be ignored. Although NSC23766 has a good effect in the treatment of resistance to EGF-TKI in NSCLC patients, it cannot be widely used in clinical treatment due to its excessively high IC50 (50 Um) (206). With the discovery of new functions of Rac1 in other diseases, the treatment targeting Rac1 is undoubtedly a promising treatment option. Therefore, there is an urgent need to develop more new Rac1 inhibitors which can be widely used in clinical treatment (**Figure 5**).

## Conclusion

Rac1, as a cytoskeletal regulator, regulates the polymerization of actin and promotes the migration and invasion of tumor cells, thus playing a key role in tumor evolution. Rac1 is highly expressed in different types of tumors, and it is related to poor prognosis. In addition, Rac1 is not only involved in tumor cell cycle, apoptosis, proliferation, invasion, migration, and angiogenesis, but also involved in the regulation of tumor cell stemness, thus promoting the occurrence of tumors. In addition, Rac1, as an entry point to explain the mechanism of drug resistance, also plays a key role in anti-tumor therapy and participates in immune escape mediated by the tumor microenvironment. Rac1 inhibitors have good prospects in the prevention and treatment of tumors. Therefore, Rac1 is considered a potential target for the prevention and treatment of cancers.

## Author Contributions

JXL and LO contributed to drafting and editing the manuscript, shared the first authorship. QL and YZ designed, revised, and finalized the manuscript. SR, YH, XL, and PY participated in the drafting and editing manuscript. JGL, LX, and JH participated in the revision and coordination. ST, LT, and QP contributed to the literature search. YT participated in the conception and coordination. All authors contributed toward data analysis, drafting, and revising the paper and agreed to be accountable for all aspects of the work. All authors contributed to the article and approved the submitted version.

## Funding

This work was supported in part by grants from the following sources: the National Natural Science Foundation of China (81972636, 81872281, 81772842), the Natural Science Foundation of Hunan Province (2020JJ5336, 2019JJ40175, 2019JJ40183 2018JJ1013), the Research Project of Health Commission of Hunan Province (202109031837, 20201020), China Hunan Provincial Science and Technology Department (2018SK7005), Ascend Foundation of National cancer center (NCC2018b68), and Supported By Hunan Cancer Hospital Climb Plan (ZX2020001-3, YF2020002) and By the Fundamental Research Funds for the Central Universities of Central South University (2019zzts832, 2019zzts833).

## Conflict of Interest

The authors declare that the research was conducted in the absence of any commercial or financial relationships that could be construed as a potential conflict of interest.

## Glossary

**Table:**

5-FU	5-fluorouracil
ARHGAP24	Rho GTPase activating protein 24
AD	Alzheimer’s disease
BC	Breast cancer
CSCs	cancer stem cells
ccRCC	clear cell renal cell carcinoma
CRC	colorectal cancer
CYRI/FAM49B	CYFIP-related Rac1 interacting protein
CSCC	cervical squamous cell carcinoma
CC	contraceptive centchroman
CLL	chronic lymphocytic leukemia cell
circRSF1	circRNA-RSF1
DADS	diallyl disulfide
DA-MED	deacetylepoxydiene
DSG2	Desmoglein-2
ESCC	esophageal squamous cell carcinoma
Erα	estrogen receptor α
EOC	epithelial ovarian cancer
ECs	endxothelial cells
EMT	Epithelial-mesenchymal transition
EPS8	epidermal growth factor receptor pathway substrate 8
GSLC	glioma stem-like cells
GEFT	Guanine nucleotide exchange factor T
GDIs	guanine nucleotide dissociation inhibitor
GAPs	GTPase activator protein
HCC	hepatocellular carcinoma
HSC	hepatic stellate cells
HNSCC	head and neck squamous cell carcinoma
HSCs	hematopoietic stem cells
ISC	intestinal stem cells
IQGAP1	IQ-guanosine triphosphatease-activating protein 1
ITGA5	Integrin alpha5
ICIs	Immune checkpoint inhibitors
IR	radiation therapy
IL17A	interleukin 17A
LIMK1	LIM kinase 1
LFA-1	lymphocyte function-associated antigen-1
MET	mesenchymal-epithelial transformation
M1-Exos	M1-type macrophage-derived exosomes
NAC	neoadjuvant chemotherapy
NPC	nasopharyngeal carcinoma
NSCLC	non-small cell lung cancer
NBS	nucleotide-binding site
OIP5	OPA interacting protein-5
OSCC	oral squamous cell carcinoma
OXA	oxaliplatin
PPP	pentose phosphate pathway
PBR	multi-base region
PGC	primary gallbladder cancer
PKC-ζ	protein kinase C-ζ
PAKs	p21 activated kinase
pp70s6k	The 70 kDa S6 kinase complexes
PLS1	Plastin1
PTPσ	protein tyrosine phosphatase-sigma
PLPPR1	phospholipid phosphatase-related protein 1
RIII	radiation-induced intestinal injury
RILD	radiation-induced liver disease
Rac1	RAS-related C3 botulinum toxin substrate 1
RCC	renal cell carcinoma
RCC2	regulation of chromosome condensation 2
ROCK2	Rho-linked kinase 2
RMS	rhabdomyosarcoma
Sema3F	semaphorin-3F
Sema3C	Semaphorin-3C
S1PR1	Sphingosine-1 phosphate receptor 1
SDF-1α	stromal cell-derived factor 1-α
SCC	squamous cell carcinoma
Tiam1	T lymphoma invasion and metastasis protein 1
TNBC	triple-negative breast cancer
TNFAIP8L2/TIPE2	tumor necrosis factor, alpha-induced protein 8-like 2
UA	Urobilin A
VEGF	vascular endothelial growth factor
VEGFR	vascular endothelial growth factor receptor
